# Effect of H_2_O on the Pressure-Induced Amorphization of Hydrated AlPO_4_-17

**DOI:** 10.3390/molecules24162864

**Published:** 2019-08-07

**Authors:** Frederico G. Alabarse, Boby Joseph, Andrea Lausi, Julien Haines

**Affiliations:** 1 Elettra Sincrotrone Trieste, Basovizza, 34149 Trieste, Italy; 2 GdR IISc-ICTP, Elettra Sincrotrone Trieste, Basovizza, 34149 Trieste, Italy; 3 ICGM, CNRS, Université de Montpellier, ENSCM, 4095 Montpellier, France

**Keywords:** pressure-induced amorphization, H_2_O insertion, high pressure

## Abstract

The incorporation of guest species in zeolites has been found to strongly modify their mechanical behavior and their stability with respect to amorphization at high pressure (HP). Here we report the strong effect of H_2_O on the pressure-induced amorphization (PIA) in hydrated AlPO_4_-17. The material was investigated in-situ at HP by synchrotron X-ray powder diffraction in diamond anvil cells by using non- and penetrating pressure transmitting media (PTM), respectively, silicone oil and H_2_O. Surprisingly, in non-penetrating PTM, its structural response to pressure was similar to its anhydrous phase at lower pressures up to ~1.4 GPa, when the amorphization was observed to start. Compression of the structure of AlPO_4_-17 is reduced by an order of magnitude when the material is compressed in H_2_O, in which amorphization begins in a similar pressure range as in non-penetrating PTM. The complete and irreversible amorphization was observed at ~9.0 and ~18.7 GPa, respectively, in non- and penetrating PTM. The present results show that the insertion of guest species can be used to strongly modify the stability of microporous material with respect to PIA, by up to an order of magnitude.

## 1. Introduction

Studies have shown that the incorporation of guest species in zeolites has been found to strongly modify their mechanical behavior [1] and their stability with respect to amorphization at high pressure (HP) [2]. In microporous materials with pore diameters from 3 to 8 Å, the superhydrated state under HP has been the subject of particular attention [3,4,5,6,7,8]. Guest species have been shown to strongly modify the structure stability and elastic properties of the studied zeolites. On insertion, the incorporation of CO_2_ or Ar in the silicious zeolite, silicalite, deactivates the pressure-induced amorphization (PIA) process and stabilizes the crystalline phase to pressures up to 25 GPa [2]. In silicalite, further evidence was found for the link between amorphization and flexibility and for strong interplay between framework geometry, flexibility and the physical properties of zeolites [9]. Increases in a material’s bulk modulus and the deactivation of PIA by incorporation of molecules indicated the existence of a coupling between mechanical properties and adsorption of guest molecules [2,10].

More recently, in the zeolite-type hydrated aluminophosphate AlPO_4_-54•*x*H_2_O (VFI structure, space group *P*6_3_), it has also been shown that the use of non- or penetrating pressure transmitting media (PTM) strongly changes the material’s mechanical properties. AlPO_4_-54•*x*H_2_O possesses a 1-D pore system along the *c* direction, in which H_2_O molecules form a disordered hydrogen-bonded network [11]. While its dehydrated phase undergoes to pressure-induced phase transition starting at 0.8 GPa, its hydrated phase shows PIA beginning at 2.0 GPa [12,13]. In H_2_O, the material undergoes superhydration effects and a decrease in the onset of amorphization from 2.0 in non-penetrating PTM to 0.9 GPa in penetrating H_2_O PTM is observed [12,13]. The insertion of H_2_O in the pores hinders pore collapse at lower pressures. Upon increasing pressure, instead of stabilizing its structure with respect to PIA, the material undergoes amorphization due to a chemical reaction at its 4-fold coordinated Al site, by incorporating 2 H_2_O molecules [8].

**Figure 1:**
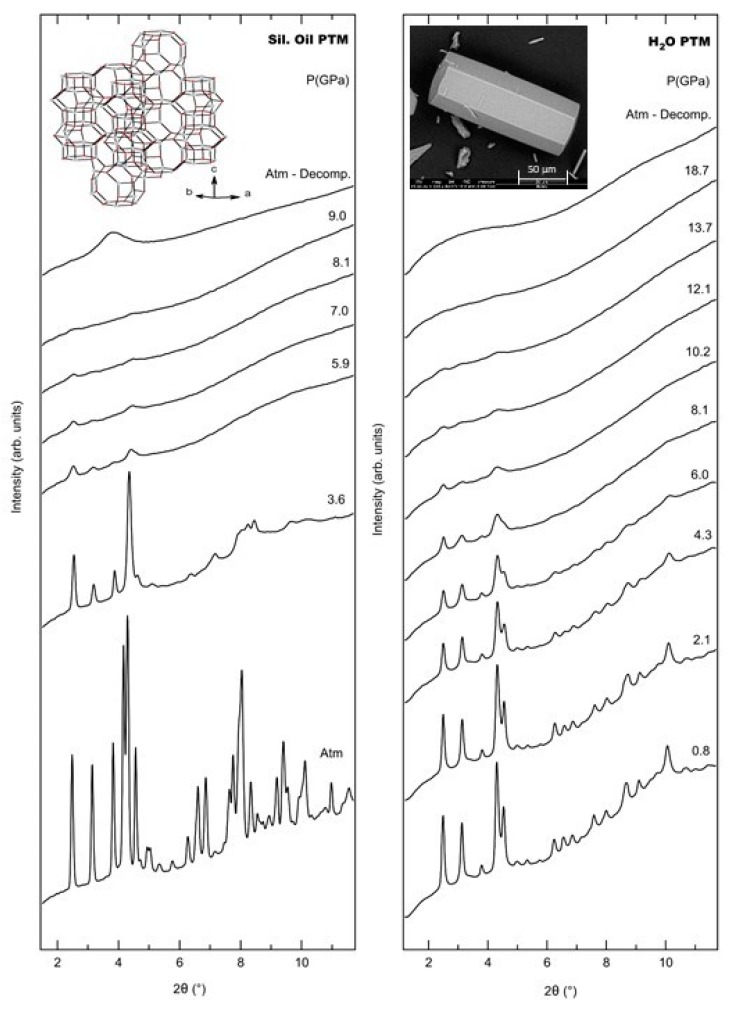
In situ powder synchrotron X-ray diffraction XRD patterns of AlPO_4_-17•*x*H_2_O in silicone oil (**left**) and in H_2_O (**right**) at selected pressures. Insert: Scanning Electron Microscope image of the as-synthesized hydrated AlPO_4_-17 (**right**) and 3D framework structure of AlPO_4_-17 (reproduced from Ref. [16]) showing the columns of alternating cancrinite cages connected along the *c* direction by hexagonal prisms forming the t-eri pore structural unit (**left**).

Negative thermal expansion (NTE) has been linked to thermally excited rigid unit vibrational modes. Such materials exhibit unusual behaviour upon compression due to the softening of a large number of these modes leading to PIA. Both properties, NTE and PIA, confer unique physical properties to these materials for several technical applications, such as support for optical components and shock wave absorption. The microporous aluminophosphate AlPO_4_-17 has an erionite structure (ERI), space group *P*6_3_/*m* and cell parameters *a* and *c* of 13.1113 Å and 15.3600 Å, respectively, and its structure is built up from rings with 4- and 6 alternating AlO_4_ and PO_4_ tetrahedra that are linked to form sheets that contain rings of 6 AlO_4_ and PO_4_ tetrahedra, cancrinite cages and rings of 12 AlO_4_ and PO_4_ tetrahedra which form the t-eri pore structure [14,15,16], see inset on Figure 1 left. Anhydrous AlPO_4_-17 has been found to exhibit a very strong NTE coefficient, the highest known for an oxide, of −35.1 × 10^−6^ K^−1^ over the temperature range of 18–300 K [14]. In the dehydrated state, all Al and P atoms are in 4-fold coordination. Very recently, the pressure effect on dehydrated AlPO_4_-17 was studied in powder samples by angle-dispersive X-ray diffraction (XRD), mid- and far-infrared (IR) spectroscopy by using diamond anvil cells (DAC) [16]. The study evidenced the mechanism of amorphization of the material: upon increasing pressure, the closure of the (P−O−Al) angle which destabilizes the porous (t-eri pore structure) of AlPO_4_-17. The material was found to begin to amorphize near 1 GPa. Both structural and vibrational analysis evidenced pressure-induced framework softening and complete irreversible amorphization near 2.5 GPa, which corresponded to the collapse of the pores. It was reported that the material initially undergoes to isotropic compression and while the lattice parameter *c* decreases nearly linearly and, atypically, the lattice parameter *a* decreases more rapidly with further increases in pressure until complete amorphization [16]. The bulk modulus and its first pressure derivative was found to be *B*_0_ = 31.2 GPa and *B’* = −10.1, respectively. The material was shown to be extremely compressible and exhibits an elastic instability. Such anomalous (negative) values of *B’* are very rare and have been observed previously in other few materials, such as cyanides (as Zn(CN)2) and metal-organic frameworks [17,18]. This instability appears to be characteristic of materials, which exhibit strong NTE [19] and indicates a link between NTE and anomalous compressibility behaviour [16].

In its hydrated state, AlPO_4_-17•*x*H_2_O (with *x* = 1.9 at ambient pressure and temperature conditions), *P*2_1_/n space group, the structure is formed by alternating AlO_6_, AlO_4_, and PO_4_ polyhedra, in which one-third from the Al sites are octahedrally coordinated with 2 additional H_2_O molecules in its coordination sphere in the CAN cages [15]. In the present study, we report the strong differences when using non- and penetrating PTM on the response of the hydrated AlPO_4_-17 structure to pressure. Unexpectedly, its hydrated phase showed a similar structural response to pressure of its anhydrous phase in non-penetrating PTM. The study was performed on the recently commissioned HP diffraction beamline from the Elettra Sincrotrone Trieste (Elettra), *Xpress*, which was developed from a partnership between the Indian scientific community administered through the Indian Institute of Science, Bangalore and the Elettra Sincrotrone Trieste, and its features will be briefly described here.

## 2. Results

After several loading attempts of the DAC, the material was found to start to become amorphous during the sample preparation stages, in particular during the grinding process. In order to avoid this problem, a loading with a less finely ground sample was necessary, which results in the final powder sample being mixed with some micron sized single crystals (SC). The initial a, b and c cell parameters of the P2_1_/n structure of AlPO_4_-17•*x*H_2_O were 13.367(3) Å, 14.908(3) Å and 22.703(5) Å, respectively, and β of 90.65(2)° at ambient pressure and temperature, and are in agreement with previous work [15]. Figure 1 shows a series of XRD patters of AlPO_4_-17•*x*H_2_O obtained in the DAC in both PTMs. The most intense reflections located at small angles, between 2.3° and 4.7°, Figure 1, such as 101, 111, 020 and 121, located at, respectively, 2.49°, 3.13°, 3.78° and 4.54°, become progressively weaker and broader in both PTMs. At 9.0 and 18.7 GPa all the reflections disappear in, respectively, silicone oil and H_2_O.

**Figure 2:**
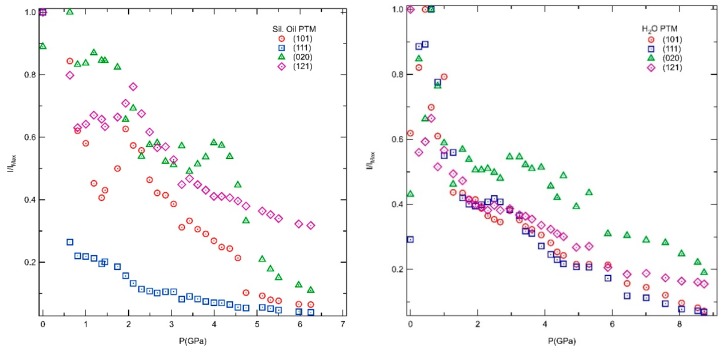
Normalized intensity (I/I_Max_) of the 101, 111, 020 and 121 reflections of AlPO_4_-17•*x*H_2_O as a function of pressure in both studied pressure transmitting media (PTM): silicone oil (**left**) and in H_2_O (**right**).

**Figure A1:**
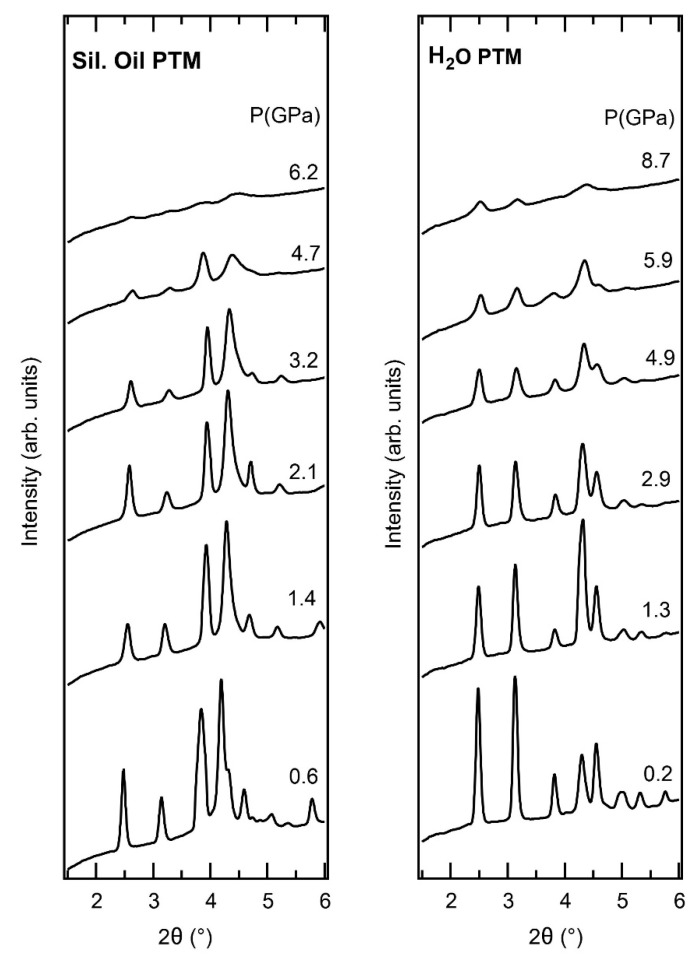
In situ powder synchrotron XRD patterns of AlPO_4_-17•*x*H_2_O in silicone oil (**left**) and in H_2_O (**right**) at selected pressures.

Figure 2 shows the evolution of the intensities of selected XRD lines as function of the pressure. When silicon oil is used as PTM, the 111 reflection seems to be the most affected at lower pressures. While some of them present significant changes up to ~1.4 GPa. Based on the rapid decrease in intensity of several XRD lines, the material starts to amorphise in this pressure range. Then at 2.1, 3.2 and 4.7 GPa differences in the slope are observed. In contrast, in H_2_O, the pressures where the diffraction lines showed significant changes in slope were at lower pressures of 1.3 and 2.9 GPa, followed by a similar decrease up to 4.9 GPa. Again, the rapid decrease in intensity up to 1.3 GPa indicate the onset of amorphization. Figure A1 (in Appendix A) shows a zoom on the most intense reflections located at small angles at the respective pressures previous described for each PTM. Above such pressures, a slower decrease in intensity for all the XRD lines are observed up to 8.7 GPa. In H_2_O PTM, the material amorphises at higher pressures, due to H_2_O insertion on the pores, which hindered the pore collapse.

**Figure 3:**
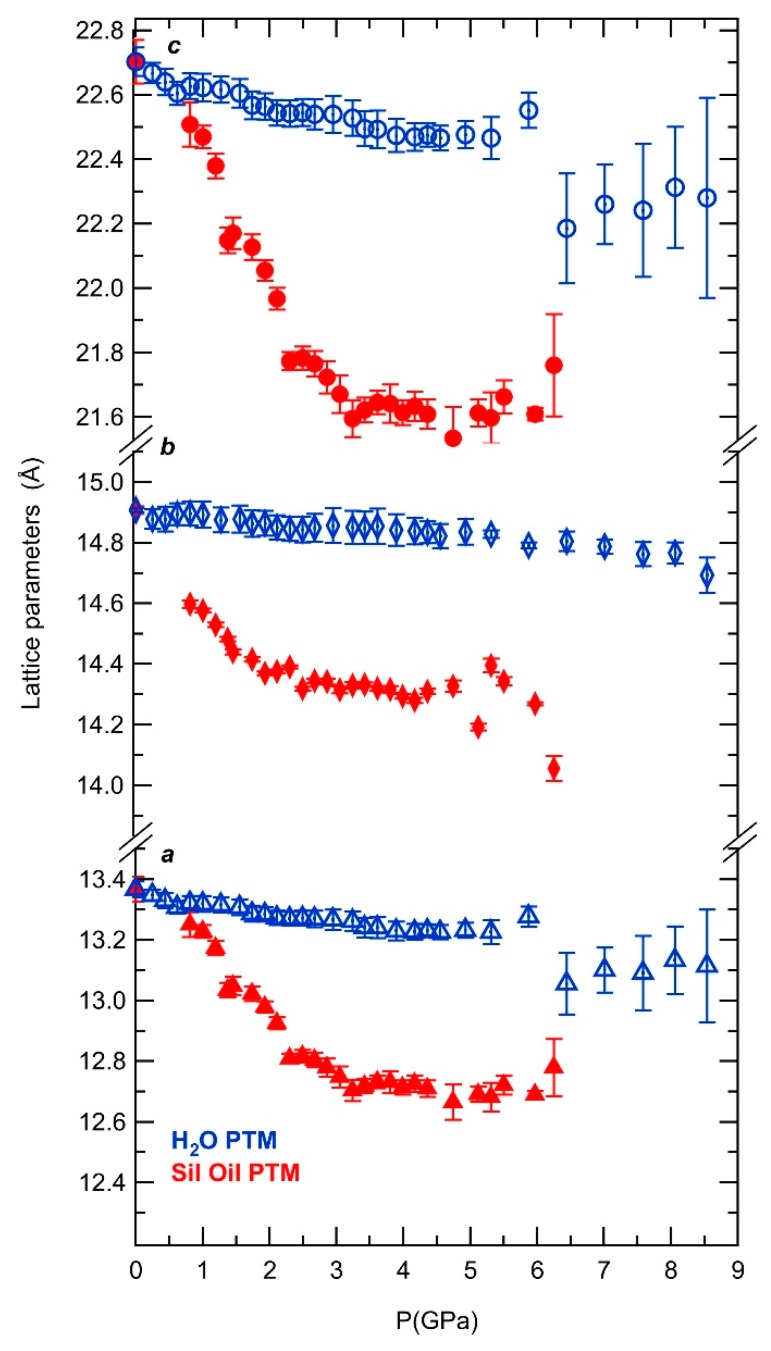
Lattice parameters as a function of pressure in silicone oil (red) and in H_2_O (blue) PTM.

Figure 3 shows the evolution of the lattice parameters upon increasing pressure. The structural response to pressure using non- or penetrating PTM is very different. Up to ~1.4 GPa using the non-penetrating PTM, silicone oil, the *a* and *c* parameters decrease more rapidly with further increases in pressure, with a contraction of 2% for *a* and *c* parameters and of 3% for the *b* parameter. This anisotropy persists up to 3.2 GPa. The *a* and *c* parameters are representative of the large t-eri pore structure, while the columns of alternating cancrinite cages (t-can) and hexagonal prisms (t-hpr), formed by two rings of 6 alternated AlO_4_, AlO_6_ and PO_4_ poly- and tetrahedra are connected along the *b* direction (the pore direction). The observed decreases are nearly linear with different slopes up to and above ~1.4 GPa. The structural behavior observed at lower pressures for AlPO_4_-17•*x*H_2_O is similar to its dehydrated phase [16], their cause and implications will be discussed later (see *Discussion* on next section). From ~3.2 GPa all parameters present a constant evolution with pressure. Strong pore collapse is observed in silicone oil in the low pressure region below 2 GPa. In the penetrating PTM, H_2_O, all lattice parameters decrease upon increasing pressure at much slower rate compared to when silicone oil is used. At ~6.4 GPa, a discontinuity in both *a* and *c* parameters evidenced strong collapse from the pore structure. Above ~5 GPa on non-penetrating media, cell parameters *a* and *c* present typical behavior of a microstructure with mixed crystalline-amorphous sample. At this pressure range, part of the sample becomes amorphous and local depressurization occurs leading to an apparent increase of the cell parameters. Such effect has been observed on other structural studies performed on zeolites under high pressures [13,20,21] in which local depressurization effects were reported due to important volume changes during the amorphization with the external pressure not being transmitting to the remaining crystalline material.

**Figure 4:**
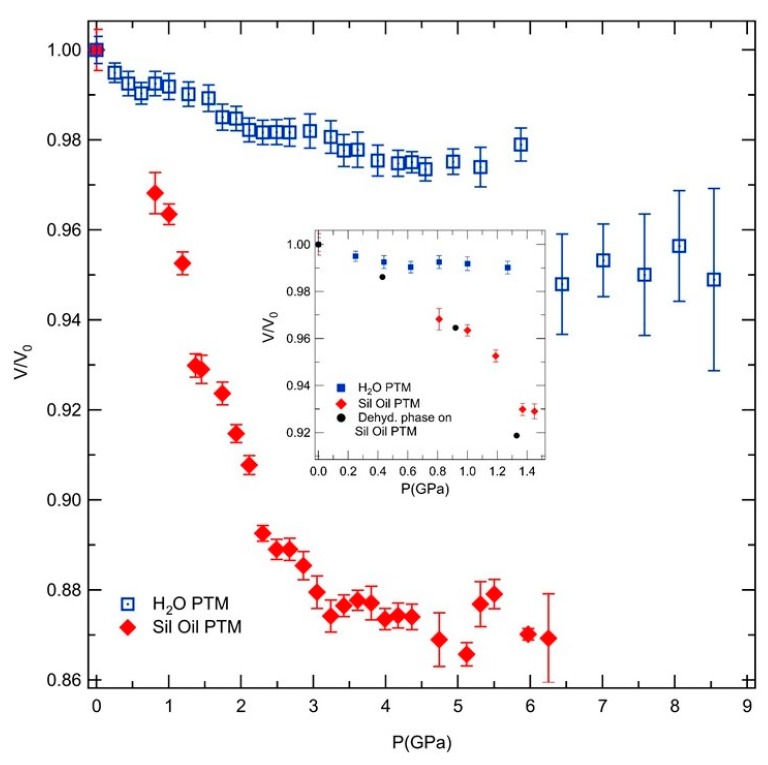
Relative volume as a function of pressure in silicone oil (red) and in H_2_O (blue) PTM. Insert: behavior on the low pressure range and (black) comparison with its dehydrated phase (reproduced from Ref. [16]).

Figure 4 shows the evolution of the relative volume (*V/V*_0_) as a function of pressure for both PTMs used. Again, the very different response to pressure of AlPO_4_-17•*x*H_2_O structure is observed when using non- and penetrating media. Whereas in H_2_O, the material exhibits a very low compressibility, in silicon oil the framework compresses rapidly by 7% at 1.4 GPa, 11% at 2.3 GPa and 13% at 3.2 GPa. In contrast, in H_2_O PTM, the structure compresses by only 2% up to 3.2 GPa. In the insert of Figure 4 we compare at lower pressures AlPO_4_-17•*x*H_2_O to its dehydrated phase. Both materials in a non-penetrating PTM present very similar compressibility at lower pressures.

## 3. Discussion

The similar structure flexibility to its anhydrous analogue in non-penetrating PTM indicates that the hydrated AlPO_4_-17 becomes more compressible as the pressure increased and that, as in the dehydrated phase, exhibits an elastic instability. Such behavior is the opposite to the structural changes as a function of pressure observed in other zeolites, which exhibit pressure-induced stiffening of the structure [22,23,24]. As evidenced by the evolution of the relative volume with pressure, there are at least four pressure ranges with different compressibility values before completely amorphization, and from which the three first, up to 3.2 GPa, seems to present pressure-induced softening, which decreases as the pressure increases. In other zeolites which exhibit strong NTE, siliceous faujasite, anomalous compressibility behavior is also observed [6,19]. As discussed in previous studies, the lattice distortions induced by pressure in the hydrated AlPO4-17 structure may be related to low-energy lattice vibrations, which should be at the origin of the observed exotic behavior under pressure [16,25], such could be described as the result of transverse vibrations of 2-coordinate O atoms bridging Al and P atoms (the Al-O-P linkages) [16]. In its anhydrous phase, these vibrations are responsible for the high NTE behavior in this material [14].

As in the anhydrous phase, AlPO_4_-17•*x*H_2_O in non-penetrating PTM undergoes pore collapse prior to amorphization, a mechanism of which corresponds to pressure-induced framework softening around the t-eri structural units due to closure of the (P−O−Al) angles in the 12-membered rings [16]. In contrast, the amorphization in the case of a penetrating PTM should have a distinct mechanism. In the latter, the material presents a classical pressure-induced stiffening of the structure. Upon comparing to other microporous aluminophosphates, such as AlPO_4_-54•*x*H_2_O, for which, when H_2_O is used as PTM, complete amorphization is observed at 4.7 GPa [13], AlPO_4_-17•*x*H_2_O undergoes complete amorphization at a pressure 4 times higher, 18.7 GPa. In AlPO_4_-54•*x*H_2_O, it was observed that in H_2_O the material undergoes to superhydration effects and, instead of stabilizing the structure with respect to amorphization, as observed in AlPO_4_-17•*x*H_2_O, a chemical reaction between the inserted H_2_O molecules and the tetrahedrally coordinated aluminum occurs, which is at the origin of amorphization and/ or reaction process [8]. AlPO_4_-17•*x*H_2_O in H_2_O should collapse around its t-eri structural units, as described on the previous section, and the changing in coordination of the AlO_4_ tetrahedra is not excluded. In the present case, the opposite situation is observed as pressure induced amorphization is observed at HP when using H_2_O as a PTM as it penetrates the pores of the sample and stabilizes the crystal structure. Although, in contrast to AlPO_4_-54•*x*H_2_O, which possesses very large 1-D pores (12.7 Å), the pore diameter in AlPO_4_-17•*x*H_2_O is more than two times smaller (5.0 Å), which makes ^IV^Al less accessible to react with H_2_O molecules.

The observed structure flexibility with pressure in AlPO_4_-17•*x*H_2_O as a function of the used PTM, opens new strategies to technological applications. In contrast to the anhydrous phase, which is hydrophilic and requires special atmosphere conditions for its application, the hydrated phase can be easily handled in air. Pressures up to 1.4 GPa are routinely surpassed industrially, which here corresponds to a contraction of the material of 7% of its initial volume. The phenomenon of PIA has important implications for shock-wave absorption, as needed for containment for the nuclear industry and in the field of energetic materials safety. A material presenting NTE is of interest of industries such as aerospace and telecommunications, where the precision of optical components can be severely affected by variations in temperature. Combining both properties in a one single material would be highly desirable, even more as no special atmospheric conditions are required, in contrast to its dehydrated analogue.

## 4. Materials and Methods

### 4.1. AlPO_4_-17•xH_2_O: Sample and Experiment

Single crystals (SC) of an aluminophosphate with a ERI framework topology were synthesized by using *N*,*N*,*N*′,*N*′-tetramethyl-1,6-hexanediamine as a template and distilled water by a sol-gel procedure followed by hydrothermal treatment based on the optimization of methods already described [16]. SC of AlPO_4_-17•*x*H_2_O, with maximum dimensions of 250 × 70 × 70 μm^3^, were synthesized, see insert on Figure 1 right. In order to eliminate the amine, the product was calcined in air at 500 °C for 24 h. After calcination and rehydration in air, the products remained monoclinic with ERI structure (space group *P*2_1_/n), and has initial *a*, *b* and *c* lattice parameters of 13.367(*3*) Å, 14.908(*3*) Å and 22.703(*5*) Å, respectively, and β of 90.65(*2)* ° at ambient pressure and temperature [15].

In situ synchrotron X-ray diffraction (SXRD) measurements under HP were performed by using a membrane diamond anvil cell (MDAC, Almax-Plate) in angle dispersive mode. Silicone oil and H_2_O were used as non- and penetrating PTM, respectively, along with a ruby as a pressure gauge. The pressure was determined based on the displacement of the R_1_ and R_2_ of ruby fluorescence lines [26]. Two distinct pressure runs were performed for each PTM, while the first using large (2–3 GPa) the second made use of small pressure steps (0.2–0.4 GPa), Figure 1 and Figure A1 (Appendix A), respectively, presenting the most interesting results. The sample, gently ground SC, the PTM and pressure gauge were placed in an Inconel alloy gasket with a hole size of 175 × 60 µm (diameter x thickness), loaded in a MDAC with 550 µm diamonds culets size of Ia type and Boehler-Almax design with an 85° 4θ X-ray opening. Typical exposures times were of about 20 s. SXRD patterns were obtained with large 2D image plate detector, MAR345, using a 100 µm pixel size. The incident wavelength of 0.4957 Å and a 80 μm pinhole was used to provide the collimated incident beam (see next Section 4.2. for beamline description). CeO_2_ powder was used to calibrate the instrument by using the Fit2d software calibration tool [27], giving the distance between the sample and the detector of 287.93 mm. The intensities were integrated as a function of the diffraction angle 2θ to obtain a conventional one-dimensional diffraction pattern using the Fit2d software [27] with further treatment performed with the Dioptas 0.4.0 software [28]. The program Fullprof [29] was used to refine the unit cell parameters. The Xpress experimental hutch contains both a ruby fluorescence spectrometer and an automatic pneumatic pressure controller online. Both coupled with a translation rail, allow fast and reproducible measurements and positions from the beam to the PRL position. In the present study, it leads to 30 and 35 SXRD patterns-pressure points (from the ambient up to the higher studied pressure and pressure release to the atmospheric conditions), respectively, for the experiment in non- and penetrating PTM, performed both (including loadings and sample preparation) in 3 effective experiment shifts (one shift = eight h).

### 4.2. The Xpress Beamline

With the recent opening of the Xpress beamline the HP diffraction community have a dedicated experimental set up at the Elettra synchrotron facility. This new beamline is part of a scientific partnership between India and Italy under a project administered through the IISc Bangalore, comprising also the macromolecular XRD beamline *XRD2*. Both beamlines utilize the radiation from a 49 pole, 3.5 Tesla Superconducting Wiggler (SCW, Budker Institute of Nuclear Physics, Novosibirsk, Russia), Xpress employing the leftmost part of the wiggler fan to produce a 25 keV monochromatic X-ray beam (0.4957 Å) focused on a large area detector, MAR345 (marXperts GmbH, Norderstedt, Germany), for data acquisition in angle dispersive mode. This configuration is highly suitable for powder diffraction experiments to be performed under HP using a variety of DACs. The beamline station is equipped with state of the art facilities for HP manipulation: an online ruby fluorescence spectrometer (Laser 405 nm, Hamamatsu, Japan), high magnification long working distance microscope (Zeiss Discovery V.20), precision microdriller (Almax easyLab), automatic pneumatic pressure controller (General Electric, PACE5000), etc. In front of the detector, a precision motorized stage enables an easy switching between the pressure monitoring ruby fluorescence and the diffraction data collection using finite size (tens of microns in diameter) monochromatic X-ray beam.

## 5. Conclusions

The PIA on AlPO-17•*x*H_2_O was investigated in situ on powder samples at the Xpress beamline using non- and penetrating PTM. The results indicate strongly different behavior depending on the PTM used. The compression of the material is strongly reduced in the penetrating PTM, H_2_O, and the structure is stable to much high pressures. The commissioning of in situ single crystal synchrotron XRD measurements on Xpress will allow the material to be investigated in greater detail in order to understand both flexibility mechanisms and amorphization dependence on the used PTM.
